# Antenatal Depressive Symptoms and the Risk of Preeclampsia or Operative Deliveries: A Meta-Analysis

**DOI:** 10.1371/journal.pone.0119018

**Published:** 2015-03-19

**Authors:** Rong Hu, Yingxue Li, Zhixia Zhang, Weirong Yan

**Affiliations:** Department of Epidemiology and Biostatistics, School of Public Health, Tongji Medical College, Huazhong University of Science and Technology, Wuhan, Hubei, China; University of Pennsylvania, UNITED STATES

## Abstract

**Background:**

The purpose of the study was to investigate the association between depression and/or depressive symptoms during pregnancy and the risk of an operative delivery or preeclampsia, and to quantify the strength of the association.

**Methods:**

A search of the PubMed, SCI/SSCI, Proquest PsycARTICLES and CINAHL databases was supplemented by manual searches of bibliographies of key retrieved articles and review articles. We aimed to include case control or cohort studies that reported data on antenatal depression and /or depressive symptoms and the risk of an operative delivery and/or preeclampsia.

**Results:**

Twelve studies with self-reported screening instruments were eligible for inclusion with a total of 8400 participants. Seven articles that contained 4421 total participants reported the risk for an operative delivery, and five articles that contained 3979 total participants reported the risk for preeclampsia. The pooled analyses showed that both operative delivery and preeclampsia had a statistically significant association with antenatal depressive symptoms (RR = 1.24; 95% CI, 1.14 to 1.35, and OR = 1.63, 95% CI, 1.32 to 2.02, respectively). When the pre-pregnancy body mass indices were controlled in their initial design, the risk for preeclampsia still existed (OR = 1.48, 95% CI, 1.04 to 2.01), while the risk for an operative delivery became uncertain (RR = 1.01, 95% CI, 0.85 to 1.22).

**Conclusions:**

Antenatal depressive symptoms are associated with a moderately increased risk of an operative delivery and preeclampsia. An abnormal pre-pregnancy body mass index may modify this association.

## Introduction

Depression has become one of the leading causes of disease burden since early in the 21^st^ century [1]. It has been estimated that the prevalence of both major and minor depression ranges from 8.5 percent to 11 percent at different times during pregnancy through the use of self-reported screening instruments, including the Perinatal Depression Scale (EPDS), Beck Depression Inventory (BDI), and Center for Epidemiological Studies Depression Scale (CES-D), and this range is not significantly different from the prevalence of similarly aged, non-pregnant women [2].

Individual observational and analytical epidemiological studies suggest that depressive symptoms during the perinatal period may contribute to deleterious neonatal and obstetric outcomes, such as low birth weight in infants [3], preterm birth [4], decreased breastfeeding initiation [5], and a lengthened pre-delivery stay [6]. Two meta-analyses of observational studies have been performed regarding the impact of depression and depressive symptoms on neonatal and obstetric outcomes, and most of the studies use self-reported screening tools to evaluate the severity of depressive symptoms and then set cutoff scores for illustrating clinically significant depressive symptoms [7, 8]. One of the meta-analyses reported a strong association between depression and depressive symptoms during pregnancy and the risk of preterm birth and low birth weight, but there was no association with intrauterine growth restriction [7]. The other meta-analysis examined the relationship between depression and depressive symptoms during pregnancy and the risk of preterm birth, low birth weight in infants, Apgar scores at 1 and 5 minutes, gestational age, breastfeeding initiation, neonatal intensive care unit admission and preeclampsia (PE). However, only preterm births and a decrease in breastfeeding initiation were found to be significantly associated with maternal depression and depressive symptoms [8].

Maternal depressive symptoms during pregnancy have also been recognized as a possible risk factor that is associated with an operative delivery [9, 10] or PE [11, 12] when self-reported screening tools for depression has been used. Operative delivery refers to the use of obstetric forceps or cesarean section to facilitate the delivery [13], and PE is defined as high blood pressure that is accompanied by proteinuria after 20 weeks of gestation [14]. Traditionally, these obstetric outcomes are considered to be caused by physical factors. It is commonly thought that the important predictive factors for an operative delivery include age, fetal presentation, and the use of epidural analgesia [15], while the predictive factors for PE are thought to be nulliparity, a prior history of PE, and obesity [16]. Comparatively, less is known about the impact of prenatal psychopathology on these obstetric outcomes. These are important outcomes because both an operative delivery and PE are accompanied by an increased risk of maternal or fetal morbidity. Additionally, making a definite diagnosis of depression in a large obstetrical setting can be logistically difficult, although it is still important and necessary. Clinician-administered diagnostic instruments are the gold standard, although they are time consuming and staff intensive to implement; conversely, patient-rated screening instruments are easier to use but may have lower sensitivity and specificity [17]. Considering that adverse obstetric outcomes may cause significant, long-term, and negative health impacts, it would be useful to know the risk of adverse pregnancy outcomes, which can be predicted by simple patient-rated screening tools for depression at the obstetric visit.

However, the conclusions on the relationship between antenatal depression and/or depressive symptoms and operative deliveries and/or PE are inconsistent. Some evidence indicated that depression and/or depressive symptoms during pregnancy were significantly related to an operative delivery [9, 10, 18] and PE [11, 12, 17], while other studies reported that there was no direct association [3, 19–21]. Therefore, we conducted a meta-analysis to explore whether antenatal depression and/or depressive symptoms were significantly associated with an operative delivery and/or PE, and to quantify the strength of the association. Additionally, potential sources of heterogeneity were investigated, such as the study design of the included articles, socioeconomic status (SES) of the participants in each article, pre-pregnancy body mass index (BMI) (i.e., whether it was adjusted/controlled at the start of their research), antidepressant use (i.e., whether it was excluded/controlled), and the use of depression measurement instruments that have been validated against structured diagnostic interviews in pregnant women. We defined these instruments as reliable instruments, which included EPDS [22] and PHQ-9 [23], because they have been validated against structured diagnostic interviews and have acceptable sensitivity and specificity. Although the BDI has been validated for an obstetric population, it was assigned to the non-reliable group due to its lower sensitivity and specificity when compared to the diagnostic interview [24] and its poor internal and concurrent validity [25]. CES-D was assigned to the non-reliable group because it has not been validated in pregnant women [26].

## Methods

### Search Strategy and Study Selection

The databases of PubMed, SCI/SSCI, Proquest PsycARTICLES and CINAHL were independently searched from their start date to September 2013 by the first author (Rong Hu) and the second author (Yingxue Li). The keywords that were utilized include the following: ①depression or depressive symptoms or mood disorder; ②pregnancy or pregnant or maternal or prenatal or antenatal or pre-partum or ante-partum; ③preeclampsia or PE or preeclamptic or EPH toxemia or EPH complex, or EPH gestosis or gestational hypertension or GH or pregnancy-induced hypertension or PIH or pregnancy toxemia or complications of pregnancy; and ①operative delivery or surgical delivery or cesarean delivery or instrumentally assisted delivery or forceps assisted delivery or vacuum assisted delivery. Each broad item that is listed above (①②③④) was combined with “AND” or “OR” (i.e., (③ OR ④) AND ① AND ②), and a similar strategy was used in all of the databases.

Inclusion criteria included the following: 1) case-control or cohort designs; 2) an examination of the impact of depression and/or depressive symptoms during pregnancy on the incidence of an operative delivery and/or PE; 3) measurement of maternal depression and/or depressive symptoms during pregnancy; 4) available raw data, regardless of whether they were traced directly from the article, obtained from secondary data in the article or obtained from the authors. Studies were excluded if 1) they did not meet one of the inclusion criteria listed above; 2) antenatal depression measurement was pooled with other variables, such as anxiety, stress or mood disorders, or PE measurement was pooled with hypertensive syndromes; or 3) they were existing reviews, meta-analyses or hypothesis articles.

Articles were first selected through a title and abstract scanning, and then by a full-text review, according to the inclusion and exclusion criteria described above. A manual search of bibliographies of the key retrieved articles and review articles was performed to identify possible omissions.

### Data Extraction and Quality Assessment

The primary data of the included articles, as well as the quality assessment of those articles, were reviewed and extracted by the first and second author independently (Rong Hu and Yingxue Li, respectively). Extracted data included the source of the article, study design, country, SES, sample size, number of participants in the study group and the control group, method of depression measurement, results, and maternal age. In the studies that provided more than one category of depression or depressive symptoms (i.e., referred to the continuous measure of depression or depressive symptoms based on a minimal, mild, moderate or severe index), the latter three categories of depression or depressive symptoms were combined to provide an effect estimate that could be compared with the minimal scale (which was regarded as the reference group in studies that did not have a category for “not depressed”), on the premise that the categories were mutually exclusive. If the categories were not mutually exclusive, the severe category would be compared to the minimal category. The request for primary data was sent to the two authors, and one reply was received [12]. The article that did not provide a reply was then excluded from the meta-analysis for operative deliveries [27] because there was insufficient data to determine the number of subjects in each category of depressive symptoms. Although the raw data in the article that was written by Vollebregt were not directly available from the original article [21], we were able to get the raw data indirectly from a meta-analysis that was written by Grigoriadis [28]. When there was any confliction between the two reviewers, an agreement was reached through a discussion. Otherwise, the third author (Zhixia Zhang) was consulted.

The first and second authors (Rong Hu and Yingxue Li, respectively) independently performed the quality assessment of the case control or cohort studies using the Newcastle-Ottawa Scale, which uses a “star system” (with the highest score being nine stars) to judge the articles based on three broad perspectives: study group selection, comparability of the groups, and the ascertainment of the exposure or outcome of interest [29].

All of the reviewing processes were planned, conducted, and reported under the guidance of the standards of quality for reporting meta-analyses (Meta-analysis of Observational Studies in Epidemiology: a proposal for reporting) [30].

### Statistical Analysis

The association between depression and/or depressive symptoms during pregnancy and the risk for PE and an operative delivery that includes a cesarean section and an instrumentally assisted delivery was calculated by pooled odds ratios(OR) or pooled relative risk(RR), along with 95% confidential intervals, based on the study design of the included articles. Pooled OR was the exclusive effect size that could be used for combining case control studies while pooled RR provided the best estimates for combining cohort studies [31]. When there were both case-control and cohort studies within a pooled analysis for a measured outcome, pooled OR was used [31].

Inconsistency or heterogeneity across studies was quantified using the χ^2^-based Q-test and I^2^ metric [32]. When the I^2^ was larger than 50%, it comparatively indicated a severe heterogeneity among the included studies. When P_Q_ < 0.10, the heterogeneity was considered to be significant; and a random-effects model (inverse variance weighting method) was used. Otherwise, a fixed-effects model (Mantel-Haenszel method) was preferred for the meta-analysis. Sources of heterogeneity were, respectively, traced and evaluated through a meta-regression (restricted maximum-likelihood estimator analysis) and subgroup analyses for both of the outcomes, which included study type, SES of the participants, adjusting for pre-pregnancy BMI and the use of a reliable depression measurement, and antidepressant use.

A funnel plot was constructed and Begg’s test was performed to assess possible publication bias. P < 0.05 was considered predictive of a statistically significant publication bias. All of the statistical analyses were performed using Stata, version 12.0 (Stata Corporation, College Station, Texas, USA).

## Results

**Fig 1:**
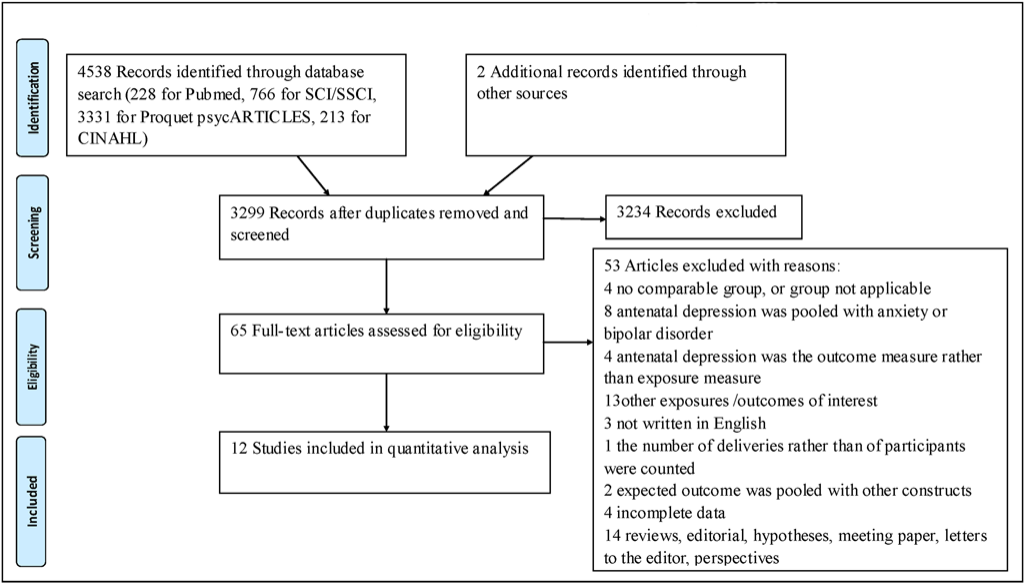
PRISMA 2009 Flow Diagram for Identification of Studies for Meta-analysis.

According to the search strategy, 3299 records were reviewed based on the titles and abstracts after 1241 duplicates were removed. Regarding the screened records, 3234 were excluded due to there being no report on the risk of an operative delivery and PE that was imposed by maternal depression and/or depressive symptoms during pregnancy. In total, 65 studies were preliminarily identified and were further assessed for eligibility through a full-text review. After a full text review, 12 studies were found to meet the inclusion criteria and 53 studies were excluded for different reasons. The detailed review process is shown in Fig. 1. Of the studies that we could pool, seven cohort studies reported on operative deliveries [9, 10, 19, 20, 33–35], and five studies reported on PE, three of which were cohort studies [12, 21, 36], and two of which were case-control studies [11, 17]. All of the identified studies used self-reported screening instruments for depression measurement. Other characteristics of the included studies can be seen in Table 1, Table 2 and Table 3. All of the included studies were given scores of six stars or above and the detailed quality assessment results are shown in Table 4. All of the results of the statistical analysis can be seen in Table 5 and Table 6.

**Table 1:** Characteristics of the Included Studies.

Article	Study design	Country	Maternal age (y) (mean/range) Study vs. control (p value)	SES	Sample size	Study group	Control group	Depression measurement	Results
Wang et al., 2010 [20]	Cohort prospective	Taiwan, China	≥20	Mixed	460	166	265	EPDS≥10	Cesarean section: 56.1% for depression vs. 30.9% for non-depression (p = 0.819); Instrumental assisted deliveries: 59.5% for moderate depression vs. 33.3% for non-depression (p = 0.785)
Imran et al., 2009 [19]	Prospective study	Pakistan	24.8 ±4.10/(18–35)	Low	176	73	103	EPDS≥12	Cesarean section: n = 34 for depression vs. n = 38 for non-depression
Bae et al., 2010 [9]	Prospective study	Korea	33.65±3.66 vs. 33.84±3.22 (p = 0.607)	Mixed	84	33	51	BDI≥10	Cesarean section: n = 15 for depression vs. n = 9 for non-depression
Wu et al., 2002 [35]	Cohort study	United States	32.0 vs. 32.9 (p<0.1)	Mixed	1697	264	1433	CES-D ≥16	Cesarean section: 26.5% for depression vs. 23.6% for non-depression; Assisted vaginal delivery: 8.0% for depression vs. 10.4% for non-depression (p = 0.34)
Chung et al., 2001 [10]	Cohort prospective	Hong Kong, China	29±4.8/(17–40)	Mixed	642	180	462	BDI ≥14.5	Operative deliveries: 39% for depression vs. 27% for non-depression (p = 0.02)

Abbreviations: SES = Socioeconomic Status, EPDS = Edinburgh Perinatal Depression Scale, BDI = Beck Depression Inventory, CES-D = Center for Epidemiological Studies Depression Scale

**Table 2:** Characteristics of the Included Studies.

Article	Study design	Country	Maternal age (y) (mean/range) Study vs. control (p value)	SES	Sample size	Study group	Control group	Depression measurement	Results
Larsson et al., 2004 [34]	Cohort prospective	Sweden	16–46	Mixed	518	248	259	EPDS ≥10	Instrumental delivery: 6.9% for depression vs. 8.5% for non-depression (p = 0.489); Acute cesarean delivery: 9.3% for depression vs. 5.4% for non-depression (p = 0.094); Elective cesarean delivery: 4.4% for depression vs. 3.5% for non-depression (p = 0.579)
Lancaster et al., 2010 [33]	Cohort study	United States	28.1±5.6 vs. 29.7±5.5 (p<0.01)	Mixed	844	159	685	CES-D ≥16	Assisted vaginal delivery: n = 38 for depression vs. n = 145 for non-depression; Cesarean delivery: n = 48 for depression vs. n = 184 for non-depression (p = 0.39)
Kharaghani et al., 2012 [12]	Case control	Tehran	28.5±5.9 vs. 27.9±5.3 (p = 0.28)	Low	312	156	156	PHQ-9 ≥ 4	Number of depressive women: n = 113 in PE vs. n = 92 in non-PE

Abbreviations: SES = Socioeconomic Status, EPDS = Edinburgh Perinatal Depression Scale, CES-D = Center for Epidemiological Studies Depression Scale, PHQ-9 = Patient Health Questionnaire-9, PE = Preeclampsia

**Table 3:** Characteristics of the Included Studies.

Article	Study design	Country	Maternal age (y) (mean/range) Study vs. control (p value)	SES	Sample size	Study group	Control group	Depression measurement	Results
Kim et al., 2013 [17]	Cohort retrospective	America	24.6±5.4/(18–44)	Unspecified	254	25	229	EPDS≥10	Number of depressive women: n = 14 in PE group vs. n = 72 in non-PE group
Vollebregt et al., 2008 [21]	Cohort prospective	Netherland	29.9±5.1 vs. 31.6±5.0 (p<0.05)	Unspecified	2114	399	1715	Dutch version of the CES-D	PE: n = 16 in depression (high) vs. n = 64 in non-depression (low)
Qiu et al., 2007 [11]	Case control	Peru	27.0±7.1 vs. 25.7±5.8 (p = 0.01)	Low	676	339	336	PHQ-9 ≥ 4	Number of depressive women: n = 159 in PE group vs. n = 123 in non-PE group
Kurki et al., 2000 [36]	Cohort prospective	Finland	≥15 or ≥30	Mixed	623	185	438	Finnish modification of BDI ≥ 3	PE: n = 14 for depression vs. n = 14 for non-depression

Abbreviations: SES = Socioeconomic Status, EPDS = Edinburgh Perinatal Depression Scale, BDI = Beck Depression Inventory, CES-D = Center for Epidemiological Studies Depression Scale, PHQ-9 = Patient Health Questionnaire-9, PE = Preeclampsia

**Table 4:** Quality Assessment of the Nine Included Studies.

	Newcastle-Ottawa Scale*									
**Author**	**1**	**2**	**3**	**4**	**5A**	**5B**	**6**	**7**	**8**	**Total**
Wang	Yes	Yes	Yes	Yes	Yes	Yes	Yes	No	Yes	8
Imran	Yes	Yes	Yes	Yes	No	No	Yes	No	Yes	6
Bae	Yes	Yes	Yes	Yes	No	Yes	Yes	No	Yes	7
Wu	Yes	Yes	Yes	Yes	No	No	Yes	No	Yes	6
Chung	Yes	Yes	Yes	Yes	No	No	Yes	Yes	Yes	7
Larsson	Yes	Yes	Yes	Yes	No	Yes	Yes	No	Yes	7
Lancaster	Yes	Yes	Yes	Yes	No	No	Yes	Yes	Yes	7
Kharaghani	Yes	Yes	No	Yes	Yes	Yes	No	Yes	Yes	7
Kim	Yes	Yes	Yes	Yes	No	Yes	Yes	Yes	Yes	8
Vollebregt	Yes	Yes	Yes	Yes	No	No	Yes	Yes	Yes	7
Qiu	Yes	Yes	No	Yes	Yes	No	No	Yes	Yes	6
Kurki	Yes	Yes	Yes	Yes	Yes	Yes	No	Yes	Yes	8

*1 indicates case definition or ascertainment of exposure; 2, representativeness of the cases or exposed cohort; 3, selection of controls or non-exposed cohort; 4, description of the control source or demonstration of outcome of interest in cohort; 5A, based on the most important factor to select and study controls; 5B, based on a second crucial factor to select and study controls; 6, assessment of exposure or outcome by blinded interview or record; 7, same method of ascertainment used for cases and controls or adequate follow-up period; 8, evenly distributed non-response rate or adequacy of follow-up of cohorts

**Table 5:** Subgroup analysis of the pooled effect of antenatal depressive symptoms on operative deliveries.

Group	No. of studies	Sample size	RR(95%CI)		Heterogeneity			Meta-regression		
			M-H pooled^1^	I-V pooled^2^	I^2^ (%)	Q_(df)_ within	P value	I^2^_res (%)	Adjusted R^2^ (%)	P value
**Total**	7	4421	1.24 (1.14, 1.35)	1.28 (1.09,1.51)	68.9	19.29	0.004			
**Socioeconomic status**										
Low	1	176	1.26 (0.89, 1.80)	---	---	---	---	68.90	0	0.946
Mixed/unspecified	6	3401	1.24 (1.14,1.35)	1.29 (1.08, 1.55)	74.1	19.28	0.002			
**Study type**										
Cohort study	7	4421	1.24 (1.14, 1.35)	1.28 (1.09, 1.51)	68.9	19.29	0.004	---	---	---
Case control study	0	---	---	---	---	---	---			
**Adjusted for pre-pregnancy BMI**										
Yes	1	1697	1.01 (0.85, 1.22)	---	---	---	---	56.48	39.65	0.172
No	6	2724	1.33 (1.22, 1.46)	1.35 (1.15, 1.58)	56.5	11.49	0.043			
**Reliable depression measure**										
Yes	3	1154	1.39 (1.22, 1.58)	1.43 (1.26, 1.62)	19.0	2.47	0.291	59.86	9.00	0.573
No	4	3267	1.17 (1.05, 1.30)	1.25 (0.99, 1.56)	71.0	10.36	0.016			
**Antidepressant use**										
Excluded/controlled	1	1697	1.01 (0.85, 1.22)	---	---	---		56.48	39.65	0.172
Not excluded/controlled	6	2724	1.33 (1.22, 1.46)	1.35 (1.15, 1.58)	56.5	11.49	0.043			

Abbreviations: I^2^_res = residual variation due to heterogeneity; BMI = body mass index

1. Fixed-effects model.

2. Random-effects model

**Table 6:** Subgroup analysis of the pooled effect of antenatal depressive symptoms on Preeclampsia.

Group	No. of studies	Sample size	RR(95%CI)		Heterogeneity			Meta-regression		
			M-H pooled^1^	I-V pooled^2^	I^2^ (%)	Q_(df)_ within	P value	I^2^_res (%)	Adjusted R^2^ (%)	P value
**Total**	5	3979	1.63 (1.32, 2.02)	1.66 (1.29, 2.13)	15.6	4.74	0.110			
**Socioeconomic status**										
Low	2	988	1.61 (1.24, 2.08)	1.61 (1.24, 2.08)	0.0	0.28	0.598	38.05	0.0	0.653
Mixed/unspecified	3	2991	1.69 (1.15, 2.48)	1.86 (1.01, 3.42)	54.6	4.40	0.111			
**Study type**										
Cohort study	3	2991	1.69 (1.15, 2.48)	1.86 (1.01, 3.42)	54.6	4.40	0.111	42.16	0.0	0.861
Case control study	2	998	1.61 (1.24, 2.08)	1.61 (1.24, 2.08)	0.0	0.28	0.598			
**Adjusted for pre-pregnancy BMI**										
Yes	2	2426	1.48 (1.04, 2.10)	1.45 (0.92, 2.29)	36.5	1.57	0.210	36.04	0.0	0.448
No	3	1553	1.73 (1.32, 2.26)	1.87 (1.29, 2.71)	24.6	2.65	0.266			
**Reliable depression measurement**										
Yes	3	1242	1.68 (1.32, 2.15)	1.69 (1.32, 2.16)	0.0	1.77	0.413	38.05	0.0	0.653
No	2	2737	1.47 (0.95, 2.27)	1.60 (0.74, 3.46)	63.3	2.72	0.099			
**Antidepressant use**										
Excluded/controlled	0							---	---	---
Not excluded/controlled	5	3979	1.63 (1.32, 2.02)	1.66 (1.29, 2.13)	15.6	4.74	0.110			

Abbreviations: I^2^_res = residual variation due to heterogeneity; BMI = body mass index

1. Fixed-effects model.

2. Random-effects model

### Risk of Operative Deliveries

**Fig 2:**
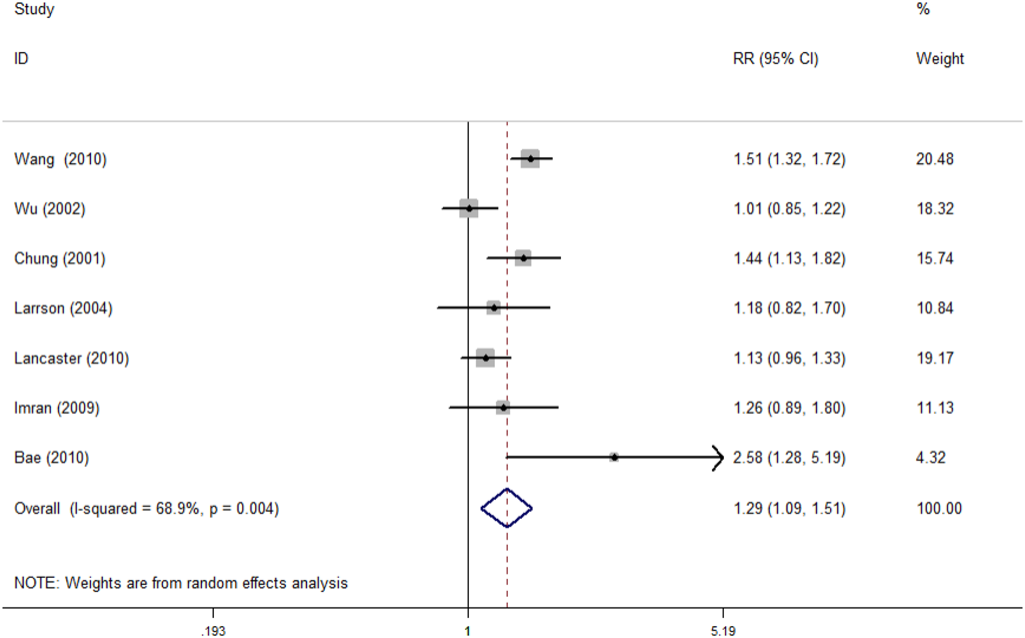
Risk of operative deliveries

The detailed statistical results are presented in Table 5. As shown in Table 5, a total of 7 cohort studies with 4421 participants were included in the investigation of the association between maternal depressive symptoms during pregnancy and the risk of undergoing an operative delivery. The pooled RR for the 7 studies was statistically significant (RR = 1.28; 95% CI, 1.09 to 1.51). Heterogeneity was detected across the studies (P_Q_ = 0.004 and I^2^ = 68.9%), and the corresponding forest plot can be seen in Fig. 2.

We conducted a univariate regression model for each possible source of heterogeneity, and no significant source of heterogeneity was found (Table 5). Furthermore, we performed subgroup analyses to evaluate the sources of heterogeneity. A stronger, statistically significant association was found in the subgroup that included studies that did not adjust for pre-pregnancy BMI, as well as in another subgroup that included studies that did not exclude antidepressant use (RR = 1.35, 95% CI, 1.15 to 1.58, and RR = 1.35, 95% CI, 1.15 to 1.58, respectively). The same results were obtained for these two subgroups of studies because the primary data that were used to pool the estimates were from the same original studies. Significant heterogeneity was also found (P_Q_ = 0.043, I^2^ = 56.5%, and P_Q_ = 0.043, I^2^ = 56.5%, respectively). The pooled RR for the SES group subanalysis was significant for the mixed and unspecified SES group, which included 6 of the 7 articles (RR = 1.34, 95% CI, 1.07 to 1.69), and heterogeneity was also found (P_Q_ = 0.003, I^2^ = 75.2%). Subgroup analyses for the study design were not performed because all of the 7 studies were cohort studies. In the subanalyses that were grouped by the use of reliable depression measures, a significant association with decreased heterogeneity was found in the “yes” group (RR = 1.39, 95% CI, 1.22 to 1.58; P_Q_ = 0.291, I^2^ = 19%).

### Risk of PE

**Fig 3:**
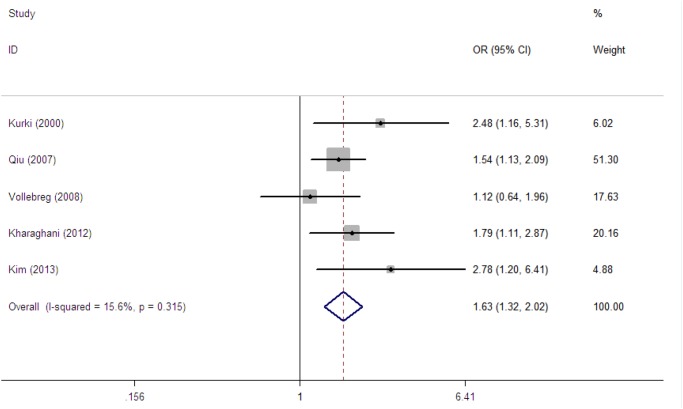
Risk of preeclampsia.

The detailed statistical results are presented in Table 6. As shown in Table 6, a total of 5 studies with 3979 participants were included to examine the association between maternal depressive symptoms during pregnancy and the risk of PE. The overall forest plot can be seen in Fig. 3. The pooled OR revealed a statistically significant association with no severe heterogeneity (OR = 1.63, 95% CI, 1.32 to 2.02; PQ = 0.110, I^2^ = 15.6%).

We conducted a univariate regression model for each possible source of heterogeneity, and no significant source of heterogeneity was found (Table 6). Furthermore, we performed subgroup analyses to evaluate the sources of heterogeneity. Subgroup analyses for whether antidepressant use was excluded/controlled were not performed because all of the 5 studies did not exclude/control for antidepressant use. All of the other subanalyses revealed statistically significant results, except in the subgroup that considered whether a non-reliable depression measure was used. No significant associations and severe heterogeneity were found in the group without a reliable depression measure (OR = 1.60, 95% CI, 0.74 to 3.46; P_Q_ = 0.099, I^2^ = 63.3%), whereas a significant association with less heterogeneity was found in the group with a reliable depression measurement (OR = 1.68, 95% CI, 1.31 to 2.15; P_Q_ = 0.431, I^2^ = 0.0%). In the subanalyses that were based on whether adjustments were made for pre-pregnancy BMI (yes/no), a stronger association with less heterogeneity was found in the “no” group (OR = 1.73, 95% CI, 1.32 to 2.26; P_Q_ = 0.266, I^2^ = 24.6%), but the association still existed in the “yes” group (OR = 1.48, 95% CI, 1.04 to 2.10; P_Q_ = 0.210, I^2^ = 36.5%).

### Publication Bias

No evidence of publication bias was found when a review of the funnel plots and Begg’s test was conducted.

## Discussion

By conducting the meta-analyses, we found that the association between antenatal depressive symptoms and the risk of an operative delivery and PE was both moderate and statistically significant. Women with an elevated level of depressive symptoms were one to two times more likely to have an operative delivery or be afflicted with PE. Our results were consistent with the results of a large hospital-based study that was performed by Bansil et al. in 2010, in which the International Classification of Diseases, Ninth Revision, Clinical Modifications was used to diagnose depression, and statistically significant associations were found between depression among pregnant women and the risk for a cesarean section or preeclampsia [18]. Our similar results suggested that not only depression that is identified by a diagnostic interview but also depressive symptoms that are revealed by self-reported screening instruments can have negative impacts on obstetric outcomes. However, the finding of there being a potential risk for PE was inconsistent with a previous review [28]. That review included four articles, and only two of them were also included in our meta-analysis [21, 36], whereas the other two were excluded due to there being unusable raw data “0” in cells that were needed to calculate OR [37] and anxiety being mixed with depression [3]. Three new studies [11, 12, 17], which were not included in the previous review, were added to our meta-analysis. Regarding the findings of a potential risk for an operative delivery in depressive pregnant women, to the best of our knowledge, this may be the first meta-analysis that has revealed this association.

In subanalyses, the risk for both an operative delivery and PE in pregnancies that are complicated by depressive symptoms was higher among studies that did not adjust for pre-pregnancy BMI at their initial design, which suggests that pre-pregnancy BMI (a recognized, common predictive factor for operative deliveries and PE [38, 39]), may modify the strength of the association. The risk for PE still existed after adjusting for pre-pregnancy BMI, which suggests there may be a clinically important association between depressive symptoms during pregnancy and PE. The risk for an operative delivery was higher among depressive women with antidepressant use. Although the nature of the outcome is not well understood, preterm birth and fetal growth restriction, which proved to be related to antidepressant use [40], may play a role in this association. The effect of antidepressant use on the risk for PE was not available in our meta-analysis. However, other studies have identified that there is an increased risk for PE among depressive women who use antidepressants [41, 42]. The association between antenatal depressive symptoms and the risk for an operative delivery and PE was statistically significant, with less heterogeneity, for those studies that used reliable depression measurements, such as the EPDS and PHQ-9; in contrast, the association was not statistically significant and revealed increased heterogeneity when non-reliable depression measurements, such as the CES-D and BDI, were used. Although the use of a reliable depression measurement was not a statistically significant source of heterogeneity, there was a trend of heterogeneity decreasing when reliable depression measurements were used. Additionally, because reliable depression measurement instruments’ identification of antenatal depressive symptoms is more similar to a clinical diagnosis of depression, this suggests there may be significant association between antenatal depression and the risk for an operative delivery and/or PE. The risk for both an operative delivery and PE for pregnant women who were afflicted with depressive symptoms was higher in mixed/unspecified SES group than in the low SES group, which was not expected. It was expected that depressive pregnant women with low SES would show a greater likelihood of having an operative delivery and PE, due to their relatively higher risk for depression [43], limited access to adequate perinatal health and mental health care utilization [44]. This contradiction may be explained by the small number of studies and small sample sizes of the studies in the meta-analyses; the true effects may not have been able to be detected. In the subgroup analyses that were divided by study type, a statistically significant association was found both in the cohort studies and the case control studies, with there being no significant heterogeneity for the association between depressive symptoms during pregnancy and PE.

In summary, our meta-analyses reveal that antenatal depressive symptoms are risk factors for an operative delivery and also for PE. Controlling for pre-pregnancy BMI and antidepressant use in studies that examine these associations appears to reduce the measured risk for an operative delivery and PE. An obstetrically validated depression measurement scale would more accurately detect depressive pregnant women. Compared to depressive pregnant women in the low SES group, the risk of operative delivery and PE was comparatively higher for those in the middle/unspecified SES group.

Although the exact psychopathophysiology that is behind the results of the associations with an operative delivery and PE has not been elucidated, we propose several possibilities. Regarding the link between depressive symptoms during pregnancy and an operative delivery, the evidence is scarce. One possibility that is proposed by Chung is that depression may impair uterine contractility, similar to the effects of anxiety, which may result in the increased risk of an operative delivery [10]. We propose that depressive symptoms during pregnancy may increases the risk of an operative delivery due to their relation to poor exercise in pregnant women because it has been reported that depression is related to poor exercise [45] and a lack of exercise is related to an increased risk for an operative delivery [10]. Regarding the link between depressive symptoms during pregnancy and PE, it has been reported that antenatal depression may negatively impact immune and inflammatory systems, thus activating vascular endothelium and resulting in PE [46]. Additionally, depression can lead to hypertension due to the altered activity in hypothalamic-pituitary-adrenal (HPA) axis [47]. Because PE and hypertension share similar characteristics, it is possible that depression may trigger similar HPA changes and eventually induce PE.

To the best of our knowledge, this is the first meta-analysis to investigate the association between maternal depressive symptoms during pregnancy and the risk of an operative delivery, for which we found a moderate and statistically significant association, which suggests the importance of detecting and preventing depression in pregnant women. Although the association between antenatal depressive symptoms and PE was not first investigated in our meta-analysis, new studies were added to extend the previous meta-analysis in this respect, and a statistically significant association was found, which suggests that further biomedical research may be required on the causal pathway for PE. Lastly, a rigorous quality assessment procedure was applied in the appraisal of the included studies which sheds light on the mediating effect of the measurement of BMI and antidepressant use, and on the effect of the use of reliable depression measurement tools.

Our meta-analysis has several limitations. Firstly, the number of included studies was relatively small, and the studies had relatively small sample sizes. Consequently, the heterogeneity across studies was prone to being incorrectly estimated as zero because the test for heterogeneity was poor at detecting true heterogeneity among studies with statistical significance [48]. Next, the pooled estimates were based on raw data with no adjustments, especially in regard to the antidepressant use data; therefore, it was difficult to identify the exclusive effects of depression on operative deliveries and PE. Additionally, variability in depression assessment was inevitable due to the differences in the type of depression (major, minor or both), and the diverse tools that were used to assess depression severity. These differences may result in a mixed level of depression severity. Although the reviewing process was performed independently and by both authors, coding was subjective, so there is the potential for error. Finally, it was not clear whether cesarean sections that were included in operative deliveries resulted from depressive women’s own choice or from their obstetricians’ preference. One study found that there were lower levels of depression among women who preferred abdominal delivery [49], while another study found a higher level of depression among patients who preferred cesarean delivery [50]. Although researches regarding obstetricians’ preference of the mode of delivery among depressive women were scarce, Chung et al proposed that health care professionals may respond to depression symptomology, consciously and subconsciously, by being more ready to opt for operative deliveries [10].

Considering the limitations that were discussed above, additional epidemiological studies are needed to obtain a more robust estimation and a better understanding of the role of depression in operative deliveries and PE. If available, larger multi-center studies that include multiracial groups with low antidepressant exposure may be essential to confirm the independent risk of antenatal depression. This would allow for more evidence-based decisions to be made to balance the number of pregnant women who are treated for depression and the number not treated for depression. Additionally, it may be necessary to differentiate between the women’s preference for cesarean sections and obstetricians’ preference for performing a cesarean section in future research so that different populations could be targeted when endeavoring to reduce the events of cesarean sections in the clinic. Further observations are needed to confirm the impact of SES. Clinicians and health policy decision makers should take antenatal depression into account during the process of antenatal health care by using an initial screening for depressive symptoms and timely and appropriate interventions for detected depressive symptoms in pregnant women to prevent severe complications.

## Supporting Information

S1 PRISMA Checklist (DOC)
